# Culturally modified trees or wasted timber: Different approaches to marked trees in Poland’s Białowieża Forest

**DOI:** 10.1371/journal.pone.0211025

**Published:** 2019-01-23

**Authors:** Tomasz Samojlik, Anastasia Fedotova, Tomasz Niechoda, Ian D. Rotherham

**Affiliations:** 1 Mammal Research Institute, Polish Academy of Sciences, Białowieża, Poland; 2 Institute for the History of Science and Technology, Russian Academy of Sciences, St. Petersburg, Russia; 3 Max Planck Institute for the History of Science, Berlin, Germany; 4 Zespół Szkół Chemicznych i Przemysłu Spożywczego im. gen. F. Kleeberga, Lublin, Poland; 5 Department of the Natural and Built Environment, Sheffield Hallam University, Sheffield, United Kingdom; Austrian Federal Research Centre for Forests BFW, AUSTRIA

## Abstract

Studies of past forest use traditions are crucial in both understanding the present state of the oldest European forests, and in guiding decisions on future forest conservation and management. Current management of Poland’s Białowieża Forest (BF), one of the best-preserved forests of the European lowlands, is heavily influenced by anecdotal knowledge on forest history. Therefore, it is important to gain knowledge of the forest’s past in order to answer questions about its historical administration, utilisation, and associated anthropogenic changes. Such understanding can then inform future management. This study, based on surveys in Belarussian and Russian archives and a preliminary field survey in ten forest compartments of Białowieża National Park, focuses on culturally-modified trees (CMTs), which in this case are by-products of different forms of traditional forest use. Information about the formation of the CMTs can then be used to provide insight into former forest usage. Two types of CMTs were discovered to be still present in the contemporary BF. One type found in two forms was of 1) pine trees scorched and chopped in the bottom part of the trunk and 2) pine trees with carved beehives. A second type based on written accounts, and therefore known to be present in the past (what we call a ‘ghost CMT’), was of 3) lime-trees with strips of bark peeled from the trunk. Written accounts cover the period of transition between the traditional forest management (BF as a Polish royal hunting ground, until the end of the eighteenth century) and modern, “scientific” forestry (in most European countries introduced in the second half of the nineteenth century). These accounts document that both types of CMTs and the traditional forest uses responsible for their creation were considered harmful to “rational forestry” by the nineteenth-century forest administration. Thus the practices which created CMTs were banned and the trees gradually removed from the forest. Indeed, these activities drew the attention of forest administrators for several decades, and in our view delayed the introduction of new, timber-oriented, forest management in the BF.

## Introduction

One of the main requirements for understanding the currently observed state of any natural environment is the knowledge of its history, and especially of past anthropogenic activities. Studying past interactions between humans and the environment is of utmost importance in the case of ancient forests with continual tree cover for perhaps several centuries. One such area is Białowieża Forest (BF), which has persisted in some form since the end of the last glaciation [1–2].

The scale, extent and meaning of anthropogenic modification of the BF are currently not only a matter of scientific interest but have been incorporated in political discussions as part of the long-term conflict around the need to protect the forest. It is crucial then to maximise opportunities to better understand the forest’s past in order to answer questions concerning its historical administration, utilisation and change. Former traditions of forest use can be and are studied in many ways. Up to the present time, different approaches were used and these include: quantification of the impact of traditional forest use, both singular, e.g. cattle pasturing [3] and cumulative [4] land-use changes in the forest in the last two centuries [5], history of anthropogenic fires in the last four centuries [6], studies on cultural landscapes created by traditional forest use [7], and the impacts of past use of individual tree species [8–9]. In many of these studies, the subject of single tree specimens affected by people historically has emerged and this provides important insights into historical and often undocumented forest use. This present study focuses on culturally-modified trees (CMTs) i.e. trees that bear anthropogenic scars or modifications that are an effect of deliberate marking or a by-product of different forms of traditional forest use. Such trees are known from other European, Australian and North American forests. In northern Sweden, for example, Scots pine trees were used for bark peeling and marking from prehistoric times until the nineteenth century by indigenous nomads (Sami) dwelling in the mountainous regions of Scandinavia [10–14]. Practices leading to creation of CMTs ranged from extraction of resources, through marking territory and trails, to the religious and magic uses of trees [12, 15]. Some CMTs, apart from scars, bore carvings that carried particular messages—in central Sweden for example, trees were often used by Swedish farmers as “notice boards” from the 1750s until the early 1900s [16]. In Australia, scarred trees and trees with dendroglyphs are considered one of the most distinctive forms of Aboriginal heritage [17–18]. Australian CMTs were analysed as evidence of Aboriginal use of bark for canoe, shield and bark shelter-making [19], and CMTs connected with the procurement of wild honey and wax allowed for analysis of indigenous patterns of landscape use before and after European colonization [20]. In North America, ancient bark-peeled trees are considered an important link to the culture of Native Americans and living artefacts of past traditional uses of the forest (traditional crafts, peeling the inner bark as an emergency food) from the mid-1600s until the early nineteenth century [21–23].

Most studies, apart from collecting information on presently-existing CMTs and analysing their chronology and spatial distribution, raise issues of their protection for cultural heritage reasons. The durability of such remnants of past forest use is generally limited by the trees’ lifespans and the additional time they can persist in dead form. Any case in which historical, archival data of CMTs are available is therefore a unique opportunity to overcome the limitations set by the life-time of a tree’s existence.

Centuries of traditional forest use created different types of CMT in BF, and these were observed and noted on different occasions since the eighteenth century. This paper explores the idea of wooded landscapes carrying the memory of past traditional uses and human-nature interactions in the form of scarred, modified or “worked” trees. Furthermore, this is in the context of introduction of modern, “scientific” forestry undertaken by the Russian administration of the Forest in the nineteenth century. The clash between the centuries-long, multifunctional use of BF and the demands of the new timber- and later game-oriented approach to “rational” forest management was observed in the case of cattle pasturing in the forest [3], and traditional beekeeping [24]. However, it also affected those types of local forest-use that are not yet widely recognized by researchers. Information collected by the forest administration in the nineteenth century offers a chance to explore those types of use which left visible traces in the form of CMTs, thus expanding our knowledge of the chronology, intensity, and spatial extent of traditional forest uses present in BF during that period.

This research aimed to collect information about types of culturally modified trees in the BF and to analyse spatial and temporal patterns of the presence of different types of CMTs in the forest. The nineteenth-century data on CMT allow quantification of the intensity of traditional types of forest use that survived in the BF even after the introduction of modern, “scientific” forestry under Russian rule. Official accounts facilitate estimation of the extent of those traditional uses and allow an assessment of the efficiency of the forest administration’s fight to control (and eventually prohibit) these types of utilisation.

## Material and methods

### Study area

Białowieża Forest, straddling an area of 1,450 km^2^, is a continuous region of temperate mixed lowland forest divided between Poland and Belarus. The Polish part, covering 600 km^2^, incorporates an area of 47.5 km^2^ of old-growth forest strictly protected since 1921 (within Białowieża National Park, covering in total 105 km^2^), and forests managed under state forestry by means of timber extraction and tree planting. The managed part is studded with smaller nature reserves with different protection regimes, but in general has a younger age class-distribution of tree stands compared with the National Park [25].

BF is considered one of the best-preserved lowland forests of temperate Europe and serves as a unique reference area for studies on ecological processes in natural conditions [26–28]. This rich, multi-species, closed-canopy forest (in Białowieża National Park– 99.2% of the study area [29]) is a mixture of five main forest types: coniferous (with dominance of *Pinus sylvestris* and *Picea abies*), mixed coniferous (*Pinus sylvestris*, *Picea abies* and *Quercus robur*), deciduous (*Quercus robur*, *Tilia cordata* and *Carpinus betulus*), mixed deciduous (*Picea abies*, *Quercus robur*, *Tilia cordata* and *Carpinus betulus*), and wet black alder bog forest and streamside alder-ash forest (*Alnus glutinosa* and *Fraxinus excelsior*). In the Belarussian part of the BF, coniferous forest dominates (69% of the area), whereas in the Polish part about half of the wooded area is dominated by conifers. There is only a small amount of open areas and this is made up of river valleys, marsh-lands, and forest gaps [25].

Białowieża Forest is one of the most valuable temperate lowland forests of Europe, with parts conserved in a state considered never modified, clear-cut, or replanted by means of modern forestry. However, though often under-appreciated, there is a long history of human presence in the forest and of its traditional, multifunctional utilisation. In ancient and medieval times, BF had several settlements with adjacent cemeteries, scattered both spatially and chronologically [30–31].

At the end of the fourteenth century, after the political union between Poland and Lithuania, BF became a royal hunting ground of Polish kings and Lithuanian grand dukes and it served this purpose until the eventual demise of the Polish-Lithuanian Commonwealth in 1795. During this royal period (fourteenth to eighteenth century), BF was excluded from any unauthorized hunts or timber-felling. Royal forest guards, foresters, riflemen and beaters were settled in villages on the border of the forest to prevent any unauthorized access [32]. Nevertheless, BF served as a source or variety of forest products. Local villagers, nobility, churches, and towns were given special permissions by Polish kings (so-called ‘access rights’) to enter specified parts of the forest and utilize them in a specific way. The traditional uses incorporated haymaking in glades and along river valleys, traditional beekeeping, fishing in rivers, and cattle pasturing [33–34]. More destructive and resource-demanding types of use, like wood-tar production, potash and charcoal burning, or commercial timber extraction, were introduced here at the end of the seventeenth century—at least two centuries later than in other Lithuanian forests [35]. In case of timber production, written sources allowed us to calculate that yearly timber extraction amounted to 0.05 to 0.3 m^3^/ha [33]. This left the BF in a well-preserved state (less than 10% of the entire area was deforested at the end of the eighteenth century) [4, 33]. After BF and part of the Polish-Lithuanian Commonwealth were seized by the Russian Empire (in three partitions of Poland, with the final one in 1795), attempts at introducing the “rational” forest management were undertaken in the late 1830s to1840s. This resulted in a clash with the traditional uses of the forest, particularly involving the persistent presence of different groups of users (herdsmen, haymakers, beekeepers, etc.) inside the forest. Throughout the entire nineteenth century, the new forest administration pursued (not always successfully) the goal of limiting or totally superseding the traditional uses, e.g. cattle pasturing, see [3]. This administrative process, apart from its actual outcome, resulted in the creation of a number of documents offering an insight into the different types of culturally-modified trees existing in BF in the nineteenth century.

### Sources of information about culturally-modified trees in BF

A survey was conducted in the National Historical Archives of Belarus in Grodno, Belarus (NHAB), the Russian State Historical Archive in St Petersburg, Russia (RSHA), and the Russian State Archive of the Navy in St Petersburg, Russia (RSAN). All references to documents in Russian and Belarussian archives are given below in compliance with established academic practice: translated title of the document is followed by original title, the abbreviated name of the archive, and the document identification number: F. (fond, collection), O. (opis, inventory), D. (delo, file), p. (page, folio). The survey yielded several previously unknown documents on traditional uses resulting in tree modifications and also on the administrative views regarding CMTs in the nineteenth century BF. Furthermore, a review of the literature was conducted concerning forest management both in the royal period (until the end of the eighteenth century) and in the nineteenth century [36–42]. This generated information on other possible forms of CMT that might not be detectable in the Forest today. All citations from archival documents and literature used in this article were translated from Polish and Russian by the authors.

For the purposes of this study, a pilot field survey to search for CMTs was conducted in spring 2017 by one of the authors (Tomasz Niechoda) in old-growth forests in the Polish part of BF (within the Białowieża National Park). Białowieża National Park has been protected since 1921 whereas most of the remaining parts of the BF have been and still are commercially exploited for timber. The selection of survey area was not random—we chose parts of Białowieża National Park where the chance of CMTs survival was considered to be the highest based on two criteria: 1) dry part of the forest accessible by foot; 2) presence of old roads and forest paths, dating back at least to the nineteenth century. Ten forest compartments (284, 285, 286, 288, 314, 316, 317, 369, 374, and 375), each measuring approximately 1 km^2^, were walked in south-north transects (10 transects in each compartment) in search of old trees carrying anthropogenic marks. All old trees were checked for scars and other marks during walking each transect. To distinguish CMTs from trees with naturally formed scars (e.g. from animal damage, fallen branches or natural fire), we were in looking for man-made features (some of which were previously known from literature and local memory of old practices that created those features): carved holes, axe cuts, stamps or other markings). Only after identifying such features, did we record the particular tree as a CMT.

Considering that the survey was carried out in an area substantially different from the rest of the forest, the goal of this part of the study was to determine the types of CMTs still present in BF, rather than estimating the number of surviving trees in the entire forest. Nevertheless, the data on the current abundance of modified trees in the ten compartments of Białowieża National Park provided a relative comparison to archival, historical data from the nineteenth century.

### GIS analysis

**Fig 1:**
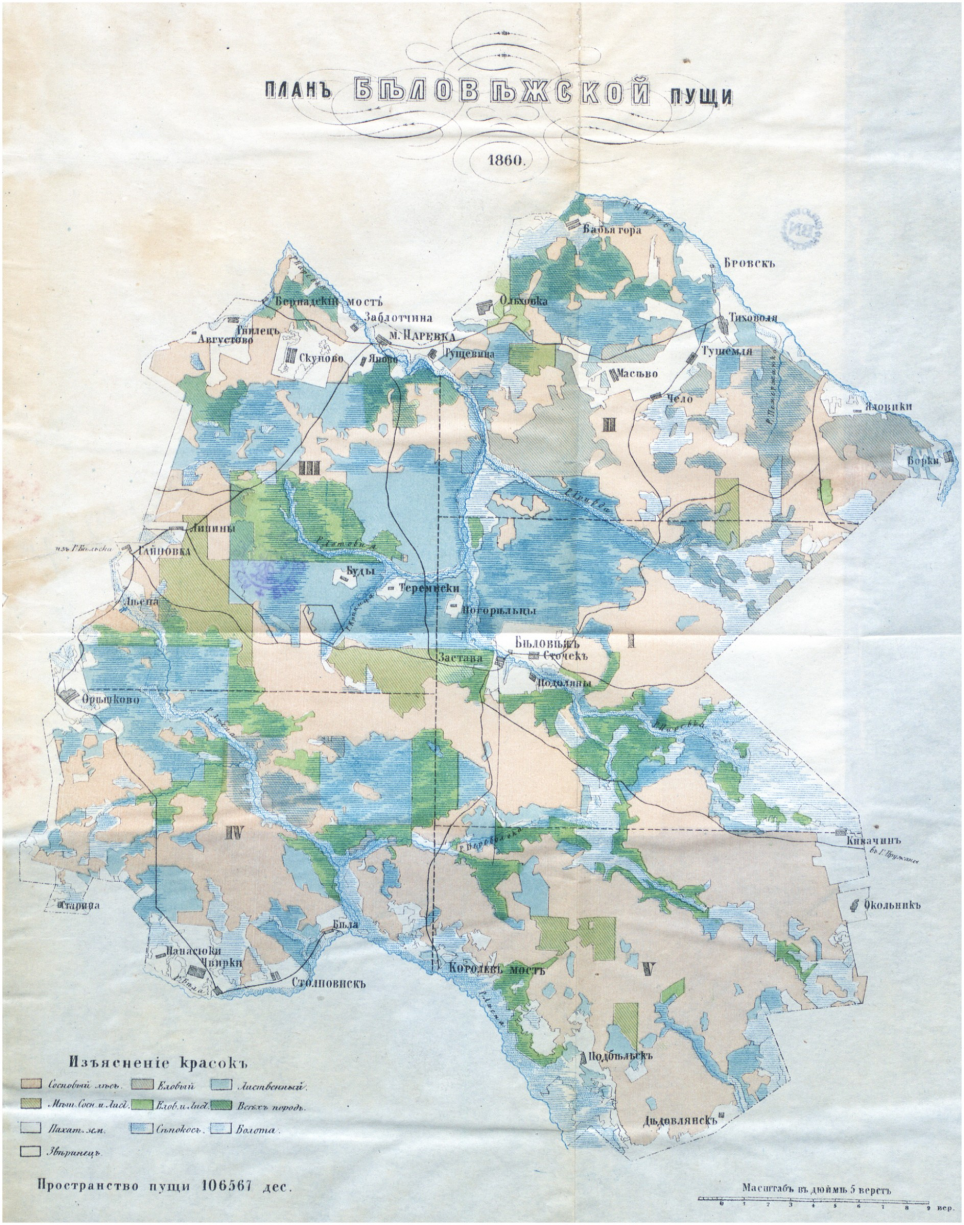
The first known map showing the forest composition of the Białowieża Forest published in 1860. Reprinted from: Bobrovskii P. Materialy dla geografii i statistikii Rossii, sobraniye ofitserami generalnogo shtaba. Grodnenskaya Guberniya. 1863; St. Petersburg: Tipografiya Departamenta Generalnogo Shtaba (under a CC BY license, with permission from Zoological Institute of the Russian Academy of Sciences, St. Petersburg, Russian Federation).

**Fig 2:**
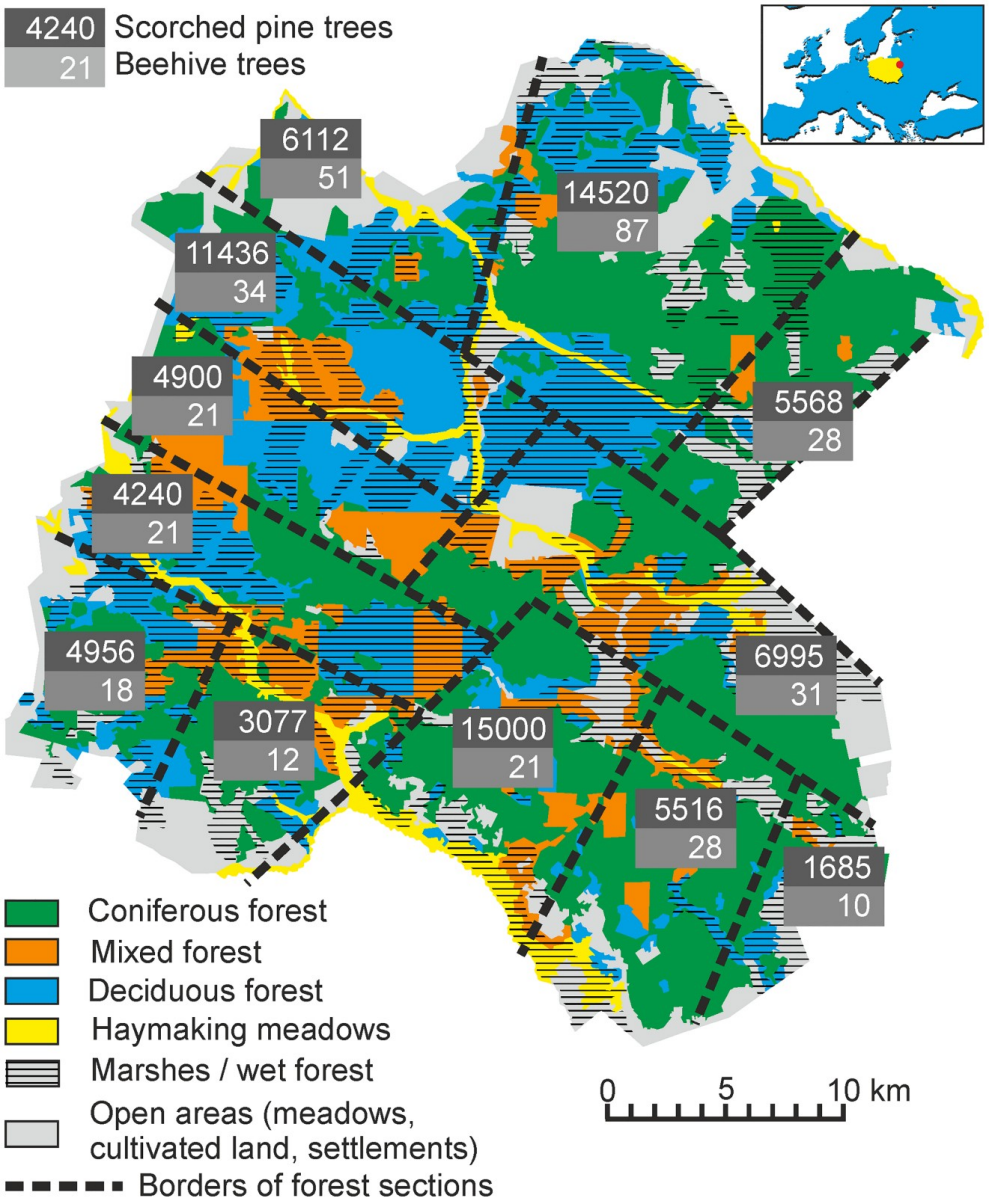
Scorched pine trees and active beehive trees in 19th-century Białowieża Forest. Based on the map from [43]. Data on scorched pine trees from 1844, and active beehive trees from 1828. Drawing by Tomasz Samojlik.

Geographical analysis of the correlation between distribution of culturally-modified trees and forest types was based on the first known map showing the forest composition of the BF published in 1860 (Fig 1) [43]. The map was an outcome of the first forest taxation conducted between 1843–1848, and depicts only limited sets of forest habitats: coniferous, deciduous, and mixed, with additional information on open areas (including settlements, forest glades and marshes), and areas for haymaking. Based on this map, the areas of different types of forest habitats and land-use were calculated (as percentages of the total area of forest depicted on the map). They were used in the analysis of the correlation between the historical abundance of CMTs and forest/land-use types (Fig 2).

Written sources concerning CMTs allowed the allocation of their numbers to twelve forest sections—administrative units, into which BF was divided until the 1840s [42]. This administrative division, dating back to the royal period, was depicted on a map published in 1830 by E. K. Eichwald in “Naturhistorische Skizze von Lithuanien, Volhynien und Podolien” [44]. The 1830 map, similar to maps from the eighteenth century [32–33], shows a separate section in the middle of the forest with Białowieża village and the royal manor. This section is not mentioned in historical sources concerning culturally-modified trees, which is strange since it contains a significant part of the forest. Another map, drawn by Jakub Sokołowski [45] presents a possible explanation. In this case, the central section with Białowieża village, is incorporated into the ninth forest unit, and this probably depicts the actual administrative division of the forest in the early nineteenth century.

Data on villages and settlements inside, on the border of the forest, and in the buffer zone of approximately one kilometre around the border, were extracted from the map “Białowieża Forest. Administrative division according to the Description of the Forest from 1796…” [36]. This map was selected as it depicts not only main villages but also small hamlets in which the forest guards dwelled. This needs to be taken into consideration when analysing possible connections between number of villages and activities that took place in the forest.

Analysis of correlation between the number of CMT, type of the forest habitat and land use, and number of villages was conducted using the Pearson correlation coefficient in R, version 3.2.3 [46].

## Results

Archival survey and literature search, along with field survey, generated information on two forms of CMTs that still exist in BF nowadays and allowed the conceptualisation of one other form of CMT. This latter form is not now present but potentially was there in the forest historically (Table 1).

**Table 1:** Types of culturally modified trees (CMTs) in BF identified in historical sources and in the field, with descriptions of their features and traditional activities connected with CMTs.

Type of CMT	Description	Activity connected with CMT	Observed currently in BF
1. Scorched and chopped trees	Pine trees scorched in the lower part of trunk with pieces of wood chopped off	Acquisition of resinous wood for kindling	Yes
2. Beehive trees	Pine trees with visible beehives, usually located several metres above ground	Traditional forest beekeeping—carving beehives in pine trees, collecting honey and wax	Yes
3. Lime trees with bark-strip scars	Lime trees with torn strips of inner bark	Bark and bast tearing from lime trees for production of bast shoes, nets, ropes, mats, baskets	No

### 1. Pine trees scorched and chopped in the bottom part of the trunk

Scots pine *Pinus sylvestris* trees were traditionally used for a source of resinous wood for kindling. The material was produced by scorching the lower part of the trunk, which caused increased production of resin in the damaged area. This effect is shown by studies on different *Pinus* species [47–48]. The wood was then chopped off and collected, and the chopped place was scorched again. This practice was probably as old as the oldest types of traditional uses described in historical sources, i.e. haymaking or beekeeping (at least since the fifteenth-sixteenth century [32]). However, it was not specifically mentioned until the eighteenth century, when collecting resinous wood was one of the entries on the price-list for different forest products: a carriage (most probably around 400 kg) of resinous wood (Polish: łuczywo smolne) was worth 20 grosz, and a carriage of smaller sticks of resinous wood (Polish: łuczywo na drzazgi)—10 grosz [36]. For comparison, the price of cheapest loaf of bread in this period amounted to 1 grosz [49]. Acquisition of resinous wood was most probably not considered very dangerous—being officially accepted by the forest administration and allowed in return for payment of a specific fee. Nevertheless, no sources were discovered that allowed quantification of this kind of utilisation in the eighteenth century.

After a short period of chaotic acquisition of BF and adjacent villages by the Russian administration, tsar Alexander’s decrees from 1802 and 1803 brought back the protection of the forest and of European bison based on a model from the previous, Polish period. For the next four decades the established traditional forest management was employed, with selective cutting in the years 1813–1821. However, from 1821, the forest, as a habitat of European bison was conserved and no timber felling allowed. It was only in 1838 that the Russian Navy started to acquire and extract oaks and pine trees for the purposes of shipbuilding [38, 42]. Misappropriation, administrative wheeling and dealing, and problems with transporting large timber forced Naval Ministry to discard plans to acquire timber from BF. In 1843, BF was taken over by the newly created Forest Department. From that point on, the focus of forest administration was to introduce a forest management plan in accordance with the modern, timber-oriented, “scientific” German forestry school [50].

Traditional use of pine trees to collect resinous wood was no longer welcome, and was prohibited. Those trees which had been used were recorded as wasted timber described as trees “spoiled in the lower part of the trunk by chopping pieces of wood” [51]. This last factor meant that this category of trees started to appear in documentation which is now very fortunate in terms of the research. This discovery offers a glimpse into the former presence of CMTs in BF in the middle of the nineteenth century. This provides insight into the landscape long forgotten and reshaped by both anthropogenic and natural processes. According to Russian reports from the 1840s, traces of use in the bottom part of the trunk were visible on numerous pine trees in the forest [41]. An inventory of such trees was carried out in 1844, and 84,005 modified trees were counted in twelve forest sections of BF (number calculated by summing numbers of trees reported for each section). The original report gave a total of 83,704 trees, a mistake in the accounts that was later repeated in subsequent letters and reports [51]). Several groups were identified as responsible for “wasting pine timber” and these included craftsmen producing wood and birch-tar, beekeepers, hay-makers scything meadows inside the forest, cattle-herders driving their beasts to forest pastures, and people passing through the forest. The head forester of the Grodno province Danilo Neuman wrote in his report that most of the damage was done in the past, yet people still collect resinous wood this way, carrying chopped pieces under clothes to bypass guard-posts on roads leading out of the forest [52]. To hide their negligence, forest guards would soot the damaged trunks themselves and explain that the damage dated back to old times. The head forester ordered them to scrape the blackened trunks to differentiate the new from the old ones. Guards and riflemen were warned that they would be removed from their posts if such situation occurred again. However, it is easy to imagine that such time-consuming and most probably futile regulations were not observed [52].

In the period between 1843 and 1846, the forest inventory team worked in BF to produce forest management plans, including location of clear-cuts and amounts of timber to be produced. Such plans were accepted by the Special Forestry Committee in Petersburg, yet before they were realized, the Grodno Chamber of the Ministry of State Domains reported that there were still 83,704 large pine trees damaged in the lower part of the trunk by chopping pieces of wood inventoried in BF. Since these trees were mostly healthy and could be still sold as timber, the Grodno Chamber proposed to exploit them immediately rather than waiting for their turn in the forest management plan. It was argued that any delay might cause their further deterioration, and soon they would be barely useful even as firewood or for wood tar production [52, 53]. In 1849–1850, the Special Forestry Committee once again reviewed forest inventory from 1843–1846 and suspended its realization, according priority to damaged pine trees [54, 55]. Thus, three years of effort by forest inventory specialists were in fact wasted and the planned felling of damaged pine trees delayed the introduction of the modern forest management to BF with its planned clear-cuts. The extent of this delay was long enough for the general attitude towards BF to change. However, a change in management emphasis now occurred with a shift towards hunting. Immediately after the first hunt by the Tsar in the forest in 1860, the forest management began to shift from timber- to game-oriented. This culminated in 1888, when BF was turned into a hunting reserve as part of the Tsar’s private properties [38].

Damaged pine trees were stamped and selected for felling in the first instance, yet this raised several problems for the forest administration. The trees were located in different parts of the BF, with no transportation routes nearby. Eventually, a merchant C.F.L. Buggenhagen from Berlin and his Russian representative S.A. Simund were contracted in 1854 to extract 60,000 pine trees “damaged in the bottom part of the trunk” in the course of the next 5 years [56]. Archival documents provide evidence that this exploitation focused rather on large-sized trees of high quality (suitable for shipbuilding, including mast trees, although felling the latter was prohibited by the contract), damaged on purpose by Buggenhagen’s workers. Furthermore, the merchant claimed that he had found only 17,200 damaged pine trees and asked for permission to cut down old but not damaged trees. Nevertheless, commissions sent to investigate charges against Buggenhagen observed that he left numerous damaged trees in the forest and estimated the number of such trees to be at least 300,000. The Naval Ministry, which considered mast trees an important strategic resource, pushed charges against Buggenhagen, but before the case was concluded, the age of wooden sailing ships had ended. From the 1860s, there was no more demand for Białowieża’s from the Naval Ministry for trees as ships’ masts [57–60].

Since Buggenhagen’s contract did not end the problems of tree destruction by established uses, forest management was still looking for ways to remove both the trees and the traditions of their use from BF. In 1861, the head forester of the province Eichwald attempted to solve the problem of damaged trees present in BF whilst at the same time satisfying the constant demand from local peasants for kindling material. By clear-cutting compartment lines in the forest (first marked in 1843–1846) [42], he planned to produce enough pine-wood to provide local peasants with resinous wood good for kindling, thus preventing further damage to valuable trees. To get rid of the meandering pathways in the forest, seen by the forest administration as harmful because they enabled fast and discrete access to the forest by poachers and other unauthorised people, straight and visible compartment lines were planned to become roads. Pine trees cut during this process were to be used as kindling material by local people instead of pieces of wood illegally chopped by themselves [61]. However, this plan did not work out, and the practice of chopping off pieces of the lower pine trunks continued until the twentieth century. Indeed, the BF’s forest administration at the time complained about herders destroying trees with this activity (along with poaching, destroying nests and carving indecent writings on poles that marked forest compartments) [62].

**Fig 3:**
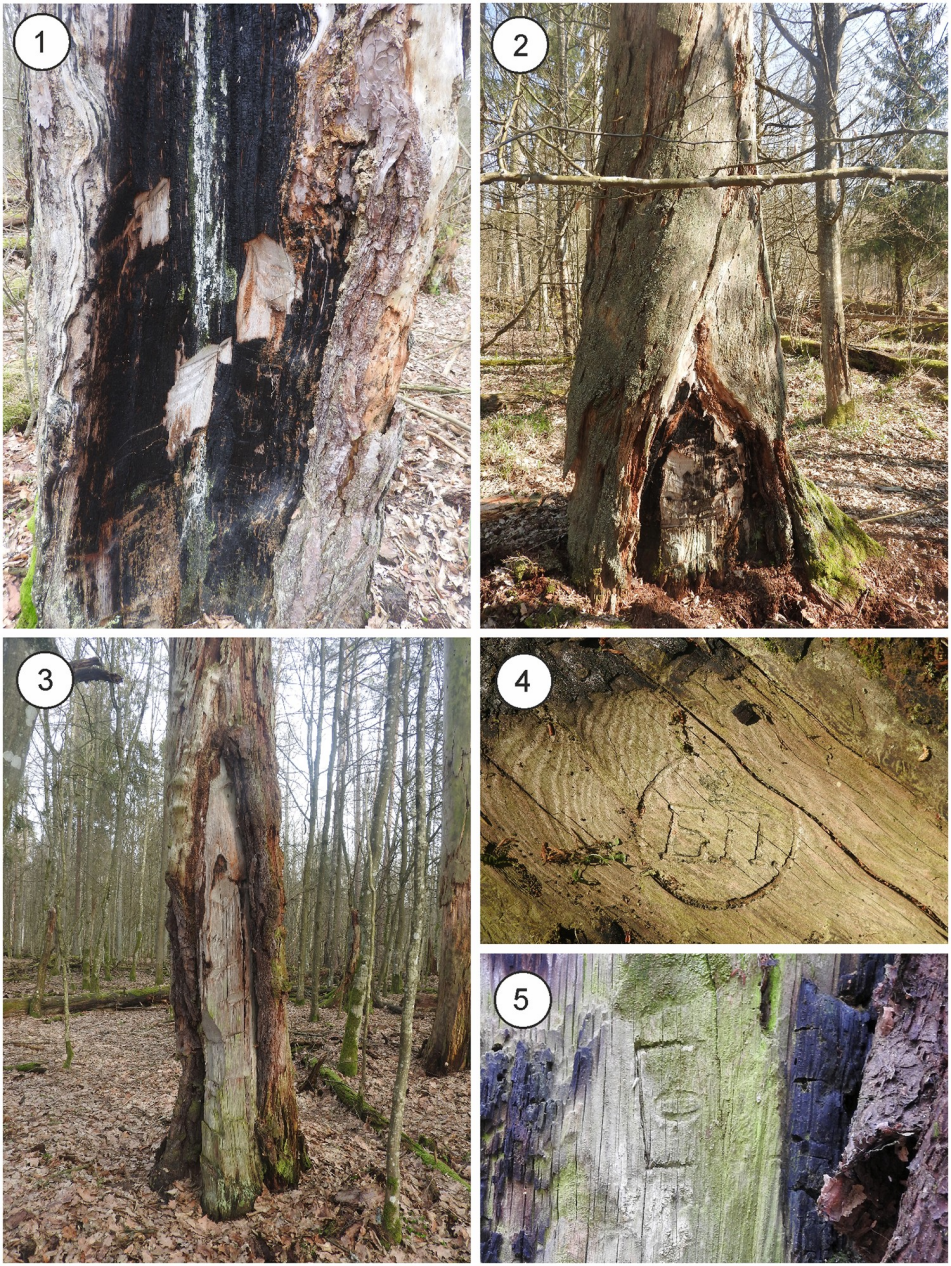
Examples of scorched and chopped CMTs still present in current-day BF. Scots pine trees scorched and chopped in the bottom part (1 & 2), only chopped, without traces of burning (3), and stamps from one of the Russian inventories of “trees destroyed in the bottom part of the trunk” (4 & 5). Photographs by Tomasz Niechoda.

Scorched pines with traces of chopping wood for kindling are still present in BF. During the preliminary survey in ten forest compartments within the strictly-protected Białowieża National Park, 286 scorched and chopped pine trees were found. These had an estimated density of 29 trees/km^2^ (most probably the density of such CMTs would be lower in the managed part of BF). Most registered trees were dead (sometimes even fallen), and most had burning marks. Only in rare cases was the chopping off of resinous wood not accompanied by scorching of the trunk (Fig 3). In three cases, there was a stamp preserved on the chopped part of the trunk—with Russian Letters “BP” (from Russian name of BF—Belovezhskaya Pushcha) or with crown and number 0 (probably designating the “zero” class of wood in damaged trees) (Fig 3).

To check for any correlation, the historical information on the distribution of scorched pine trees in twelve forest sections dated to 1844 [51] was related to data on forest habitats and land-use types from the 1860 map, and the number of villages inside and on the border of the respective forest sections (Table 2). The statistical analysis showed that the number of scorched pines did not depend on the presence of any particular type of forest, haymaking and open areas, or villages. It suggested that collecting resinous wood, although selective in terms of tree species, was not restricted geographically but was actually widespread and common for the entire BF. Based on the 1844 data, the average density of scorched pines in BF was 71 trees/km^2^, varying from 39 to 140 trees/km^2^ in different forest sections.

**Table 2:** Data on forest types (coniferous, deciduous, mixed), haymaking area and open areas in twelve forest sections inside BF derived from 1860 map [43], the number of villages within and near each section from [36] and the corresponding number of CMTs.

Forest section	No. of CMTs		Forest types (km^2^)			Haymaking area (km^2^)	Open area (km^2^)	No. of villages
	Scorched	Beehive	Coniferous	Deciduous	Mixed			
1	3,077	12	30.2	10.7	12.5	6.3	19.4	6
2	4,956	18	25.2	19.9	6.1	1.3	18.7	14
3	4,240	21	24.4	38.4	21.0	5.6	5.2	5
4	4,900	21	17.4	27.7	25.8	1.8	6.8	11
5	11,436	34	18.3	58.2	15.2	6.0	9.6	8
6	6,112	51	20.1	22.7	2.4	4.6	23.1	11
7	14,520	87	88.0	61.2	5.0	6.3	41.1	14
8	15,000	21	52.4	12.2	16.2	9.4	16.1	8
9	5,516	28	69.5	7.7	8.7	6.6	15.6	12
10	1,685	10	15.6	4.4	1.3	0.0	13.5	6
11	6,995	31	60.5	19.6	23.4	8.2	39.2	12
12	5,568	28	46.8	3.4	2.2	1.7	21.2	5
Total	84,005	362	468.4	286.1	139.8	57.8	229.5	112

### 2. Pine trees with carved beehives

Traditional forest beekeeping was the second most widespread type of forest utilisation in the period from the sixteenth until the end of eighteenth century [34]. Beekeepers, a special group of forest users whose status was hereditary and whose knowledge was passed from generation to generation, selected trees inside BF (usually Scots pine, occasionally oak and Norway spruce) to carve artificial hollows (beehives) in them (Table 1). One tree hosted one, occasionally two beehives, located on average at 7 m above ground (from 3.3 to 15 metres). Beekeepers attracted wild bees to beehives, tended them, and protected against honey- and bee-eating animals (brown bear, pine marten, forest dormouse), and once or twice per year collected honey and wax [24, 63].

In 1792, there were 936 beehives with bees and 6,219 empty ones recorded in BF, and in 1796, 632 occupied and 6,601 empty [33, 36]. After 1795, the number of active beehives (or at least active beehives for which their owners had paid the annual rent, as the data come from provincial income reports about BF), dropped drastically. The average number of active beehives from 1826 to 1837 oscillated around 322 [64], and the density of active beehive trees in this period can be estimated as 0.27 trees/km^2^. However, based on the data from the eighteenth century, when active beehives constituted from 9% to 13% of all trees with beehives, the density of such modified trees could have been ten times higher than evidenced by archival sources. Due to the archival data not representing the actual number or distribution of all beehive trees in BF, no statistical analysis was applied in this case. The number of active beehives in 1828 (the only year with precise data on the number of beehives in twelve forest sections) was used but only for illustrative purposes (Fig 2).

In 1838, the Ministry of State Domains ordered an inventory of all forest beekeeping in the state forests. This included the analysis of both positive and negative aspects of this traditional use. From the point of view of the state domains administration, the latter prevailed in most cases since negative arguments were numerous. Problems described included the low income from beekeeping that the forestry administration received, damage to trees by carving beehives, unauthorised access to the forest by beekeepers, fires caused by beekeepers, also the fact that forest beekeeping was outdated and unproductive. However, the arguments of forest beekeepers were also taken into account, and so the Minister of State Domains, Count Pavel D. Kiselev decided in the 1840s, that a transition from forest beekeeping to apiaries should be undertaken. This was to be “not in a form of strict order but as gradual, voluntary act resulting from peasants’ conviction of apiary superiority over forest beekeeping” [65]. This transitional period of tolerant attitude towards beekeeping lasted until the Emancipation Reform of 1861 in Russia, accompanied by the reform of the Ministry of State Domains. Since the ministry was also responsible for the state of serf peasants too, after their liberation it was deprived of one of its main spheres of responsibility. In 1862, the Forest Department, to reduce the administrative efforts connected with low-yielding rents, requested that the local administration of provinces discontinue all contracts allowing for use of beehives in the forests [66–67]. This regulation did not go unopposed and after numerous protest letters from beekeepers (including also letters from foresters, in many cases renting or using beehives themselves), the Forest Department postponed realisation of their decision until the 1870s [68]. In BF, the process was finalised after 1888, when the forest was incorporated into the Tsar’s private properties. At this moment, as Jan Jerzy Karpiński noted: “forest beekeeping underwent complete destruction: beekeepers were gradually removed from the forest and existing beehives were taken over by forest administration. From that time, beehives devoid of their owners and caretakers were overexploited and in an express pace met their doom” [24].

**Fig 4:**
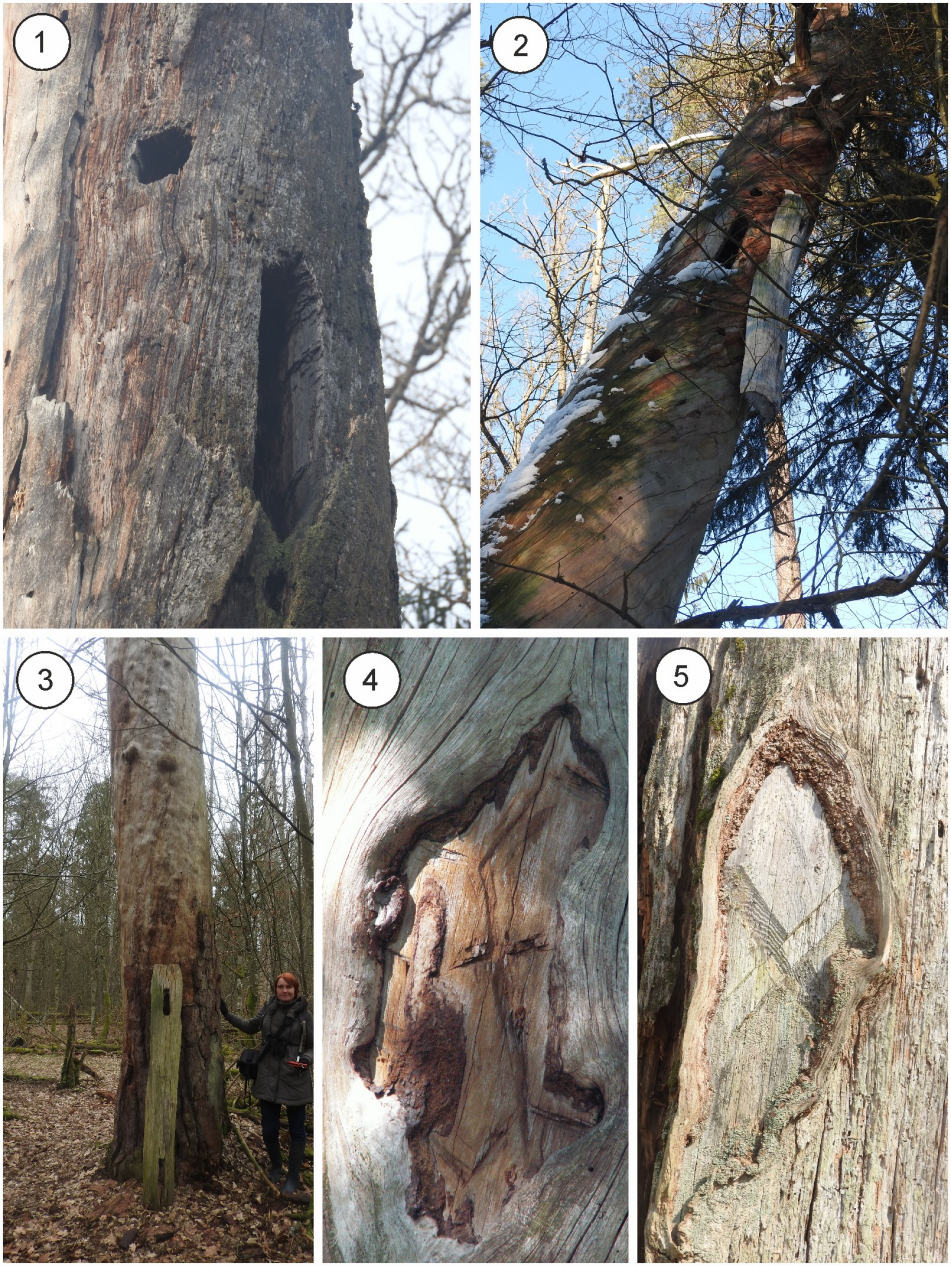
Examples of beehive CMTs still present in current-day BF. Scots pine trees with beehives (1), with construction parts—wooden plank covering the beehive (2 & 3), and ownership marks on two of such trees (4 & 5). Photographs by Tomasz Niechoda.

Apart from artificial hollows carved in the trunk, beehive trees had other characteristic modifications such as construction elements (e.g. wooden cover of the beehive and hangers hammered into the trunk), ownership marks, and protective measures against animals (e.g. sharpened poles dug at the base of the tree, wooden “collars” embracing the trunk to prevent animals from climbing the tree, and logs hung on a rope acting as a pendulum when bears pushed them while trying to get to the beehive). The first two modifications are rarely observed in the forest nowadays, as usually only the beehive hollow (sometimes overgrown by layers of new wood) survives (Fig 4). The third one is known only from the historical descriptions [24, 63].

In 1936, Karpiński described sixty-eight beehive trees in Białowieża National Park [24]. It was not a complete inventory, rather a collection of observations he made during his time in Białowieża. Nowadays, it is estimated that about 100 beehive trees are still preserved in BNP [69]. Our own preliminary survey in ten forest compartments of Białowieża National Park resulted in the finding of thirty-one beehive trees, with most being dead and in various stages of decay. In rare cases, the ownership marks are still visible (Fig 4).

Based on the historical information, another type of CMT (No. 3 below) was expected to be encountered in the forest.

### 3. Lime with strips of bark peeled

Since at least the sixteenth century, small-leaved lime *Tilia cordata* was used on an everyday basis by the local people for a variety of uses. These uses included: bast tearing (stripping of narrow strips of bast tissue from a living tree to craft different products for everyday use, like shoes, nets, ropes, mats or baskets), charcoal burning, making bee-hives, collecting flowers and seeds for medicinal use, and in producing a variety of objects from lime wood [8]. In the context of the CMT study, the first type of use, i.e. lime as a source of bast strips is the most interesting (Table 1). In the royal period in the history of BF (fourteenth to eighteenth century), various uses of lime were legally allowed. Furthermore, lime bast tearing for an individual’s own use was considered an essential right of local dwellers, confirmed in 1557 “Ustawa na wołoki” issued by King Zygmunt August [36]. In 1567, local peasants complained that the Białowieża chief forester prohibited them from gathering bast and bark, to which the royal commissioners replied that he should allow them to continue this traditional use [36]. In the seventeenth and eighteenth centuries, a special annual fee for obtaining lime bast in BF was in force. By the eighteenth century, the forest administration started to notice and report the negative impacts of this use (evidenced by, for example, a royal commission evaluating the state of BF in 1700, recommending that peasants “do not tear bast from live trees as they wither in many places”) [8]. The widespread occurrence and popularity of lime-bark tearing rose to such alarming levels that new pathways and roads were created by people entering BF for bast [8]. However, a consequence was that strengthening of forest protection was proposed in 1795, though no actual steps were undertaken and this kind of use continued also under Russian rule [36].

**Fig 5:**
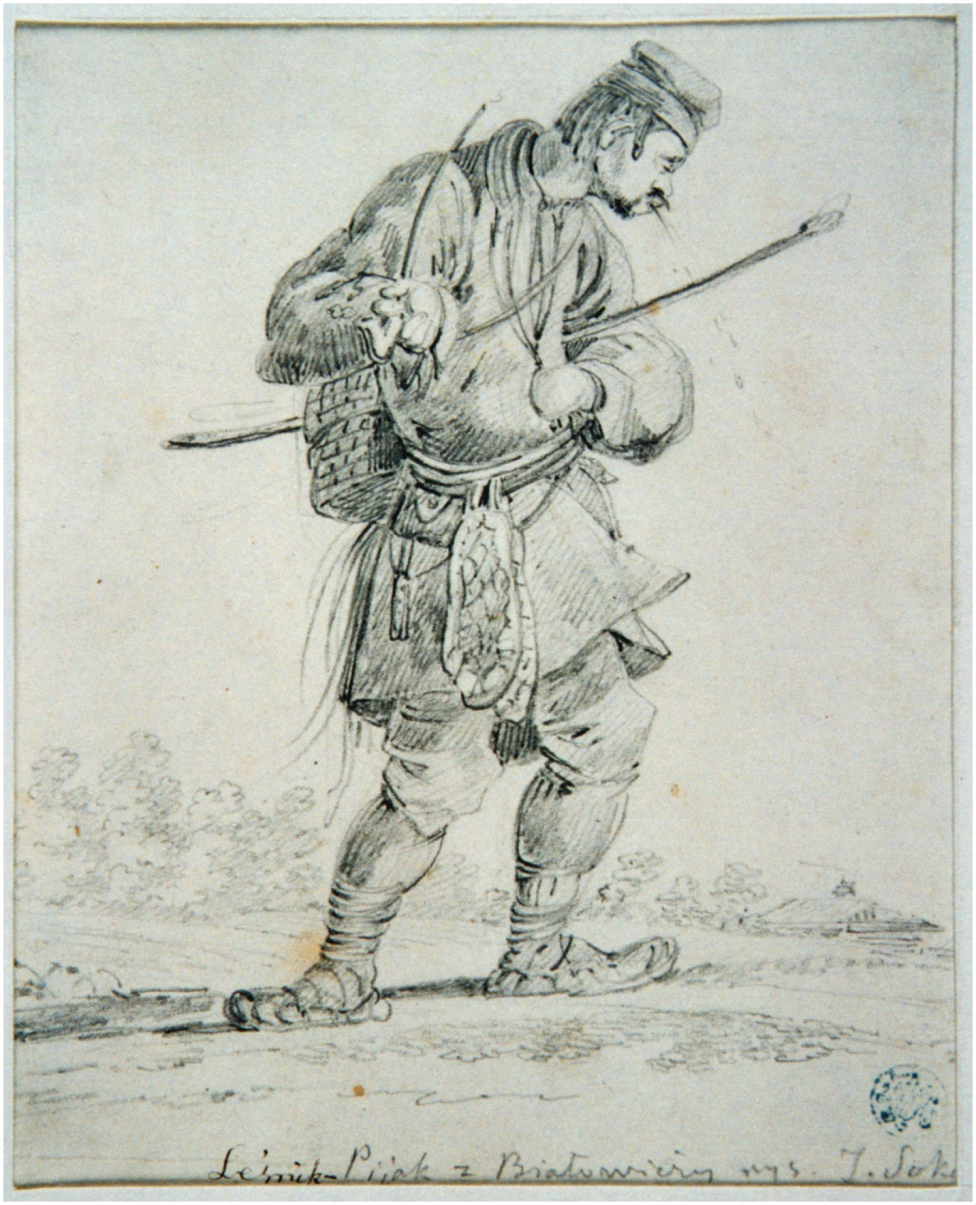
Examples of historical use of small-leaved lime bast by the nineteenth-century dwellers of BF. Bast shoes, basket, and strings of bast. “Drunk forester from Białowieża”, Jakub Sokołowski, c. 1821, pencil drawing on paper (from the collection of the Cabinet of Prints, Drawings, and Watercolours of the National Museum in Cracov, sign. III r.a. 2165).

According to nineteenth-century descriptions, two types of lime bark were stripped (in May and June): 1) large mats (usually measuring 2.5 x 1.5 metres) from the bottom part of the trunk and 2) long strips (up to 4 metres in length) from the upper part of the trunk and branches. The first type was used to produce roof covers, chests and crates, and the second to weave mats (used mainly for transportation of goods), baskets, ropes, bags and shoes [70–71]. Bast shoes were mentioned by Julisz Brincken, a prominent forest manager visiting BF in 1821, as one of the main reasons behind observed decline of lime trees: “no peasant, while being in the forest, will pass on occasion to prepare several pairs of such shoes, and even beaters make them with ease during hunts” [45]. Bast was also divided into long strands used to weave ropes, bind hay or tie various things [71]. Evidence that lime bast was widely used in the nineteenth-century BF comes also in a form of a 1821 picture drawn by Jakub Sokołowski, a Polish artist who visited BF to illustrate Julisz Brincken’s book “La foret imperiale de Bialowieza en Lithuanie” (Fig 5). It depicts a forester from BF equipped with several lime-made items including bast shoes (with a spare pair attached to his belt), bast basket, and strings of bast (which could be used to attach, pack and carry things, repair shoes or basket, weave nets, etc.).

In an 1816 report about illegal hunting by a leaseholder of Białowieża property called Pusłowski, lime bast is mentioned as one of the advantages derived from the forest without permission by his men. The report contains information about a search conducted among dwellers of Białowieża village, which revealed thirteen fresh sheaves of lime bast [72].

The importance of lime bast products was observed across much of the Russian Empire with Keppen noting in the Journal of the Ministry of State Domains (presenting the official ministerial view), that whilst peasants use a lot of lime, the resource is so important for this social class that it is impossible to restrict it [73]. In 1862, the same journal published an argument that widespread use of lime bast shoes was having less impact on Russian forests than would be the case if all shoes were made of leather. The latter, in the author’s opinion, would not only be much more expensive for peasants but also would require significant amount of wood-based (bark) material necessary in the tanning process and would render Russian export of leather impossible [74]. The same article estimated that one peasant family used 190 young lime trees annually for own purposes (among others, to make fifty pairs of bast shoes).

The central Russian administration noted the problem and in 1859 ordered an inventory of the popularity and extent of lime use among peasants in all provinces of the empire. It was explained that “manufacturing lime products allows for the poorest peasants to earn their livelihood and pay taxes” [75]. Answers came from forty-six provincial Chambers of State Domains and fifteen head foresters. In twenty-nine provinces (among them, the Grodno province including BF), “there is no craft based on lime and no disposal of the tree is therefore needed”. In a footnote, however, the author of the document clarified that this statement does not incorporate the use of lime tree in form of shrubs, “found in all forests of European Russia, bark of which is being peeled by locals (…). This lime often grows up to the size of a tree suitable for tearing large mats of bark but is not sold formally for those purposes but rather as building or heating material”[75].

Alongside the official view that there was no commercial use of lime wood in BF, other sources confirm that lime bast was being used (only for local purposes, not commercially) until the end of the nineteenth century. Tearing bast from young lime trees was reported to be such a widespread activity in the 1880s Grodno Province, that lime trees were almost absent in private forests, and were to be found only in better protected state forests (including BF) [76]. According to the report, bast shoes were the main footgear of local dwellers that used leather shoes only on special occasions. This wayward activity was characteristic especially for herders pasturing cattle in the forest: “almost every herder, returning home with the herd in the evening, brings back a stock of bast” [76]. This activity probably survived until the twentieth century with photographs of BF dwellers in an album published in 1903 showing most people wearing modern leather shoes, but some with traditional bast ones [77].

Such widespread and long-lasting peeling of lime bark might be expected to leave a visible trace in the form of culturally-modified, scarred small-leaved lime trees, yet none were found during the preliminary survey in BF. Scars on lime trees associated with peeling should therefore be considered “ghost CMTs”, and their absence in BF is discussed below.

## Discussion

Two types of CMT were discovered in BF and three identified in historical sources. Based on the results above these were: 1) scorched pine trees chopped in the bottom part of the trunk; 2) pine trees with carved beehives; and 3) “ghost CMTs” of peeled lime. All three can be treated as an evidence of centuries-old, traditional, multi-functional utilisation of the BF surviving well into the era of modern forestry. Modified trees are not the only case of historical, low-intensity forest use since cattle pasturing, for example, was present in the forest until the mid-twentieth century [3]. The most widespread type of traditional use in BF, haymaking in meadows and along forest river valleys, persisted even longer, almost to the end of the twentieth century [78]. What is unique in the case of CMTs is that their presence has most probably spanned the process of transition between the traditional and “rational”, modern forest management. Furthermore, the perceived damage they caused may have influenced forest utilisation. Management decisions in the 1840s and 1850s focused on removing modified trees (in official documents described as “damaged” or “spoiled”) from the forest and prohibiting the local use that created them; now evidenced as CMTs. It is difficult to estimate the extent to which this hindered introduction of modern forestry, but it certainly postponed some clear-cut felling highlighted in subsequent forest management plans (the first produced by 1846) [54, 55].

As described above, removal of damaged pine trees was not completed even by the early 1860s. Indeed, indicated by the remaining CMTs, the goal of removing modified trees from the forest was never achieved. Furthermore, as noted, after the Tsar’s hunt in 1860 the entire management of BF started shifting from timber production to game for the chase. This again halted attempts at introducing full-scale timber production. Without the disputes over CMTs, maybe the Russian administration would have had fewer problems in introducing clear-cuts in BF at an earlier date. In that case, it can be argued that the nineteenth- and twentieth-century history of the forest could have taken a very different route, with much earlier introduction of large-scale clear-cuts. It is likely that much of the conservation interest and cultural value of the forest found today would have been seriously compromised.

“Damaged” trees were not the only factor that hindered the introduction of modern forestry to BF: the presence of the last population of European bison (*Bison bonasus*) in BF and the Tsars’ special interest in preserving this unique animal was also. In many cases, this goal conflicted with forest management, e.g. reports that sounds of timber felling scared the bison away led to temporary stoppage of felling [38]. CMTs can be treated as a symbol of all those factors that were connected with the past, traditional function of BF as a royal hunting ground (where large game and the forest itself were guarded by a small army of royal servants [32–33]). It was also a place of multi-functional, sustainable use by generations of local dwellers [34, 78].

The fact that peeled lime tree CMTs are not today found in BF can be explained in a number of possible ways. Firstly, lime trees carrying scars from bark peeling may simply be absent from the forest because other local uses resulted in the removal of the entire tree. Bark peeling was only one of many uses this particular tree species. Indeed, all parts of the tree were used, from bark and wood, to leaves, and flowers. It might be that this intensive utilisation could have led to increased pressure on lime and the cumulative effect resulting in a decrease of the share of lime in tree-stands of BF [79]. It was observed that the lime trees present today in BF fall into two general age categories: 1) trees older than 130 years and 2) trees originating after the First World War (WW1). The gap for trees between 90 and 130 years old might evidence a period of limited lime regeneration at the end of the nineteenth and the beginning of the twentieth century. Although Konrad Wróblewski and Józef Paczoski ascribed this to overabundance of red and roe deer and competition between lime and hornbeam [80–81], it was also the last period of increased anthropogenic pressure on this species [8]. Conversely, the regeneration of lime perhaps occurred only after intensive use of the species ceased i.e. post WW1 [82–83].

Results of the analysis of the presence of nineteenth-century scorched and chopped pine trees in relation to forest habitats, land-use types and number of villages inside and on the border of the forest, are interesting. The research showed that none of these factors was decisive in determining the present spatial distribution of CMTs. This is informative and suggests that the traditional use of pine trees was common and more-or-less equally spread over the entire BF. Interestingly, even in the more coniferous parts of the forest, the number of CMTs remained similar to the deciduous areas. This might suggest that: (1) there were some mechanisms preventing overuse of certain, pine-rich parts of the forest, (2) pine chopping and scorching never reached an industrial scale, and it was not a commercial utilisation, or 3) the nineteenth-century BF generally had higher proportions of pine and spruce. Maybe these trees were abundant in all forest types.

The reliability of the historical data is always an important factor in environmental history work, and our paper is not an exception. There is a possibility of bias resulting from the selection of documents for preservation by historical actors. However, in the case of the Russian forest administration this seems unlikely for two reasons. Firstly, the administration itself was strictly hierarchical and bureaucratic, and all aspects of forest management were reported to at least two levels of higher authorities. Not reporting cases like the presence of “spoiled trees” or removing archival documentation of such on a large scale was highly unlikely. Secondly, archives visited during preparation for this work contained continuous series of documents concerning the management of BF from 1830s until the end of Russian rule over the forest in 1915, and we did not find any significant gaps in archival information in that period.

The history of CMTs in this particular Polish example illustrates processes of much wider relevance i.e. the process of disenfranchisement of local dwellers from their traditional uses of the forest. This has strong similarities to colonial processes in eighteenth- and nineteenth-century Australia and North America. There, European colonization impacted the indigenous forest management, disturbing or even entirely stopping the traditional stewardship of resources. Similarly to such cases, CMTs in BF record the prior system of land use often erased by modern management systems. CMTs are a visible, persisting evidence of different forest management regimes from several centuries ago. As such they are a cultural component of the forest landscape. On the other hand, these trees are still a part of living nature and of the natural forest life-cycle, in which competition between trees and natural dynamics accelerate the exchange of tree generations. Aging and decaying CMTs will eventually vanish from BF, but before they do, it is important to record and analyse them in order to gain insight into the forest’s history and to address questions that otherwise could not be answered based on historical sources alone.

Our findings help inform the on-going debate surrounding the management of BF (in 2017 the dispute was on the list of the most important global science events [84]). The recent outbreak of European spruce bark beetle (*Ips typographus*) followed by mass-scale logging using heavy machinery ignited the discussion on the future of the forest. Many foresters and forestry experts favour turning BF into intensively managed and regulated forest, and most of the scientific community urging the strengthening of the protection of natural processes in BF [85, 86]. One of the arguments used by foresters is the suggested tradition of forest management in BF reaching back to the beginning of the nineteenth century. This has led them to the conclusion that most tree-stands in the forest are artificial or man-made [86]. The evidence of all the trouble the forest management has had with introducing “rational” forestry to BF in the first half of the nineteenth century, along with the knowledge that from the 1860s the main role of the forest was to serve as the Tsars’ hunting ground, change this perspective. Such modern, “rational” forest management was introduced to BF only in the twentieth century (in fact after WWI), shortening the period of significant human impact on the composition of tree stands to just one century.

The culturally modified trees of BF remain an interesting topic with much still to be discovered. There are several opportunities for future research:

1. A systematic inventory of all CMTs in the entire forest (Polish and Belarussian parts).
2. More detailed analysis of spatial distribution, age and chronology of use of surviving CMTs and introduction of new methods of analysis (e.g. circular plot sampling, documenting scar morphology and photogrammetric recording of these deteriorating sites).
3. Creation of a conservation plan for the most important veteran trees with a plan for their use in education (e.g. on past, traditional and multifunctional forest management).
4. Comparison with other sites where CMTs were studied in search for common global patterns of different aspects of past life of traditional populations and their expression in the form of CMTs.
